# Feeding Practices Among Children Aged 6–36 Months Living in Urban Abidjan, Cote D’Ivoire: The Victory Cross-Sectional Study

**DOI:** 10.1016/j.cdnut.2025.107536

**Published:** 2025-08-26

**Authors:** Jeanne H Bottin, Amed Coulibaly, Stéphane Parfait Sablé, Julie Derrien, Peggy Drouillet-Pinard, Sassor Odile Purifine Aké-Tano

**Affiliations:** 1 Danone Nutricia Research, Nutrition Team, Gif Sur Yvette, France; 2 Institut National de Santé Publique, service de Nutrition, Abidjan, Côte d’Ivoire; 3 Département de Santé Publique et Spécialités/UFR sciences Médicales, Abidjan, Côte d’Ivoire

**Keywords:** children, Africa, Côte d’Ivoire, dietary diversity, dietary intake, food consumption, eating behavior, nutrition

## Abstract

**Background:**

Micronutrient deficiencies, often resulting from the consumption of diets with low nutritional value and diversity, remain a major public health concern for children in low-income countries. Understanding food consumption practices is crucial for providing an evidence-based foundation for designing and implementing effective intervention strategies to address micronutrient deficiencies in vulnerable geographies and populations.

**Objectives:**

To assess infant feeding practices and dietary intakes of healthy children aged 6–36 mo living in Abidjan, Cote Ivoire.

**Methods:**

Food and beverage intake was collected by their mother using an interview-based 24-h dietary intake questionnaire. Dietary intake was assessed after classification based on food groups and subgroups defined by the Food and Agriculture Organization/World Health Organization, adjusted to reflect Ivorian food specificities. Dietary quality was evaluated through dietary diversity scores.

**Results:**

Four hundred seven children were included (6–11 mo old: *n* = 213; 12–23 mo old: *n* = 135; and 24–36 mo old: *n* = 59; 53% female). Exclusive and nonexclusive breastfeeding prevalence at 6 mo was 37% and 95%, respectively. Breastfeeding continuation rates were 85%, 38%, and 3% in the 6- to 11-, 12- to 23-, and 24- to 36-mo-old group, respectively. The most consumed food groups were dairy (92% of the sample), cereals (88%), roots, tubers, and starchy foods (38%), fish (49%), and vegetables (41%), whereas meat (11%), fruits (15%), eggs (15%), pulses (3%), and seeds and nuts (2%) were seldom consumed. Milk intake decreased with age, whereas cereal and starchy food consumption increased. Dietary diversity was low (dietary diversity score: 3.3 ± 1.4), increasing with age. The proportion of children not meeting the minimum dietary diversity was 74%.

**Conclusions:**

The study provided information on the dietary intake of children aged 6–36 mo. The results showed that the children’s feeding practices and dietary intakes are suboptimal. This calls for interventions to improve child feeding practices in this age group.

## Introduction

Low- and middle-income countries bear an elevated burden of micronutrient deficiencies, especially in children and females of reproductive age [1]. Iron, vitamin A, iodine, folate, and zinc deficiency are the most prevalent causes for concern worldwide [2]. Micronutrient deficiencies, which contribute to poor growth, intellectual impairments, perinatal complications, and increased risk of morbidity and mortality, remain a major public health concern [[3], [4], [5], [6], [7]]. They often result from the consumption of diets with low nutritional value and low diversity as well as poor hygiene conditions, especially in low- and middle-income countries. A diet lacking in diversity may increase risk of other micronutrient deficiencies, possibly impairing children’s physical and cognitive development. The WHO guiding principles for feeding breastfed and nonbreastfed children recommend that children aged 6–23 mo be fed a variety of foods, including daily intake of animal-source food and fruits and vegetables to ensure adequate nutrient intakes [8]. This developmental period is critical both because it coincides with the peak period for risk of growth faltering and nutrient deficiencies and for children to learn to accept healthy foods and beverages and establish long-term healthy dietary patterns. A previous study by Aboagye et al. [9] exploring the association between diet diversity and undernutrition in children aged 6–23 mo from sub-Saharan countries found that having adequate minimum dietary diversity (MDD) was associated with 12% less likelihood of being stunted, 13% reduced odds of wasting among children, and significantly lowered risk of underweight by 17% compared with those who had inadequate MDD. In this study, the overall prevalence of MDD was 25.1% across all countries, ranging from 5.6% to 43.9%, with a prevalence of 11.1% for Côte d’Ivoire. In particular, the consumption of nutrient-rich foods, such as eggs, dairy products, fruits, and vegetables, is particularly important during this period to avoid increased risk of stunting. Understanding food consumption practices is therefore crucial for providing an evidence-based foundation for the design and implementation of effective intervention strategies to address micronutrient deficiencies in vulnerable geographies and populations. However, dietary intake data collection such as 24-h recalls is both burdensome and complicated in young children because of the difficulty in assessing portion sizes and leftovers, evaluating ingested food over wasted food, and estimating breastmilk quantities for breastfed children. The collection of qualitative data requires a trained interviewer and means that it constitutes a real challenge for developing countries to collect recent data on a regular basis.

Among micronutrient deficiencies, iron deficiency is the most common one, affecting more than one-third of the population worldwide and the major cause of iron-deficiency anemia (IDA) [10]. Anemia can cause fatigue, weakness, shortness of breath, and dizziness and alter cognitive and physical development in the long term [10]. In Africa, ∼60% of children younger than 5 y suffer from anemia, of whom half suffer from IDA [11]. In Côte d’Ivoire, as many as 50%–75% of children may be anemic, and iron deficiency is the most common cause of anemia in preschool children, with ≤39% suffering from IDA [12,13]. Thus, the eradication of iron deficiency has been identified as a public health priority for the country through national nutrition policies [14,15]. The main causes of IDA are the consumption of an iron-poor diet, a diet containing iron with limited bioavailability, and malnutrition—especially in periods of life where needs are increased. In this context, the 6- to 36-mo period is a crucial period because of both the exhaustion of prenatal iron reserves and the beginning of food diversification with a diet that may not contain sufficient iron-rich foods [16]. No recent study has examined the iron status of Ivorian children younger than 5 y, and to our knowledge, no dietary study has yet evaluated the iron intake of young children in Côte d’Ivoire.

To support mothers and/or health care practitioners in identifying infants at risk of insufficient iron intake, a digital tool was developed by Danone Nutricia Research with the support of Côte d’Ivoire National Institute for Public Health. The tool, called the Iron Calculator, is a simplified version of a 24-h dietary record, capturing a restricted choice of 56 foods and corresponding portions adapted for each age category and each meal. At the end of the questionnaire, the tool presents dietary recommendations to increase iron intake and absorption. The Validation of Iron CalculaTOR study (VICTORY study) was developed with the ambition *1)* to collect food consumption and nutrient intake data in 6- to 36-mo-old Ivorian children; *2)* to evaluate the prevalence of iron deficiency, anemia, and IDA in the study population; and *3)* to compare the ability of the Iron Calculator to estimate iron intake with that of a 24-h dietary intake questionnaire and to assess the ability of the Iron Calculator to preidentify children who have low iron intake, who are anemic, or who have IDA. The objective of this first set of analyses is to describe the food consumption and child feeding practices of 6- to 36-mo-old children, acquired from the 24-h dietary intake questionnaire. Further analyses relating to nutrient adequacy, iron intake, and IDA, and the Iron Calculator will be described in future research.

## Methods

### Study design and settings

This observational study was conducted using data from an interview-based 24-h dietary intake questionnaire carried out in 3 sites of Abidjan, Côte d’Ivoire between August 9 and November 4, 2022: the Infant and Maternal Health Service of the Adjamé National Institute of Public Health, the hospital in Plateau, and the Paediatrics Service at the Angré University Hospital.

The study involved a single interview carried out during a routine visit of the children and their mother at one of the sites. The study protocol (#040-22/MSHPCMU/CNESVS-kp) was approved by the National Ethics Committee of Life Sciences and Health Sciences in Côte d’Ivoire [Comité National d’Ethique des Sciences de la vie et de la santé (CNESVS)] in July 2022. There was no influence of the COVID-19 pandemic on data collection approaches. A total of 7 interviewers and 3 phlebotomists were recruited for the study. All interviewers were purposely recruited from the pool of national health workers, who are trained by the National Nutrition Program in collecting anthropometric data and collecting dietary information and follow a continuous training, with refreshers every 6–12 mo to ensure qualitative data collection. Interviewers received instructions and 2 d of refresher training for participant recruitment and data collection, followed by a practice interview exercise, which consisted of a dietary record assessment in 10 children (4 children between 6 and 11 mo and 6 children between 12 and 36 mo). A quality control resulted in the selection of 6 interviewers. Therefore, 2 interviewers and 1 phlebotomist were selected for each site. In addition, a supervisor was appointed for each site to supervise daily progress of the study and assess the quality of the 24-h dietary intake questionnaires at the end of each day. All mothers provided written or digital print consent before enrolling their child in the study, and all data were anonymized before being transmitted and analyzed.

### Survey population and sample size calculation

The population study comprised apparently healthy children aged 6 to 36 mo accompanied by their mothers. Children were not eligible if they presented with any chronic illness [such as sickle-cell disease (drepanocytosis) and β-thalassemia, among others], were hospitalized within 2 wk prior to the study start, needed urgent medical treatment, or if their mother did not know what the child consumed the day prior to the visit. Mothers were separated into either digitally-fluent mothers, if they had some knowledge in informatics, and were able to manipulate applications on a smartphone or tablet, or non digitally-fluent mothers in the other case. Children and their mothers were recruited into the study based on their eligibility when they attended the study sites. The first child recruited at each study site, and thereafter, every third child was offered to participate in the biological substudy and give a blood sample until recruitment targets were reached.

The sample size was calculated based on the comparison of iron intake between the Iron Calculator digital tool and the 24-h recall using the Bland–Altman method [17]. The anticipated mean difference in the measured iron intake between the Iron Calculator and 24-h recall is 0.47 mg/d (5% of reference nutrient intake). Assuming a standard deviation of 1.5 times the mean difference (i.e., 0.7 mg/d), a maximum allowed difference of 2.02 mg/d (20% of reference nutrient intake), 10% incomplete data, 80% power, and a 5% significance level, 399 parent–child pairs were needed for the study. Recruitment numbers in each group were estimated based on the frequentation of the study sites (54% 6–12 mo old, 33% 12–24 mo old, and 13% 24–36 mo old) and an equal distribution across the 3 study sites (Table 1).

**TABLE 1:** Distribution of children in each group according to age, study site, and mother’s digital status.

		Total initial	INSP	Plateau Hospital	Pediatrics (Angré)	Total target
Digitally-fluent^1^ mothers		81	27	27	27	81

Number of children to recruit	6–12 mo (54%)	215	72	72	72	216
	12–24 mo (33%)	132	44	44	44	132
	24–36 mo (13%)	52	18	18	18	54
	Total	399	134	134	134	402

Number of children for biological substudy	6–12 mo	40	14	14	14	42
	12–24 mo	40	14	14	14	42
	24–36 mo	40	14	14	14	42
	Total	120	42	42	42	126

INSP, Institut National de Santé Publique.

1 Digital status: mothers were separated into either digitally fluent mothers, if they had some knowledge in informatics and were able to manipulate applications on a smartphone or tablet, or nondigitally fluent mothers in the other case.

At the end of the study, the mothers received a Lucky Iron Fish (Lucky Iron Life; https://luckyironlife.com/), a small reusable iron cooking tool that infuses meals with iron to help prevent and treat iron deficiency. They also received a set of nonbranded educational cards on dietary iron developed by the authors specifically for the study.

### Data collection

Data collection was carried out during a routine visit of the child at one of the study sites. Following a screening questionnaire to assess eligibility, medical history, dietary habits, anthropometric measures, and 24-h dietary intake, data were collected by the research team from the National Institute of Public Health, Abidjan, Côte d’Ivoire.

### Anthropometric measures

The weight (to the nearest 100 g) and height (to the nearest 1 mm) of the participants were measured by interviewers during the routine visit, using an electronic scale (SECA 876) and a mechanical measuring rod. For the height (or length), children younger than 2 y or <87 cm tall were measured in a lying down position, whereas children aged 2 y and older or measure >87 cm were measured standing according to the National Nutrition Program protocol [18]. The sex-adjusted WHO weight-for-age and height-for-age curves, as well as boxplots, were used to identify outliers who were excluded from the anthropometric analyses. For the remaining children, WHO/International Obesity Task Force curves were used to define the ponderal status of the population. For children who were born prematurely (before 37 wk), a corrected age was calculated.

### Dietary intake

All mothers completed a 24-h dietary recall through a 24-h dietary intake questionnaire and the Iron Calculator. If the mother was confident in using a smartphone and mobile applications, she completed the Iron Calculator online herself without the support of the interviewer and then proceeded to complete the 24-h quantitative dietary intake questionnaire during a face-to-face interview with the interviewer. If the mother was unable to use mobile applications, she completed the Iron Calculator with the support of the interviewer and then proceeded to complete the 24-h dietary intake questionnaire with the same interviewer. Mothers were asked to recall all food and drink consumed by the child during the 24-h period prior to the interview with the support of the interviewer. Data were recorded using a paper food diary and a list of 84 preselected commonly consumed food items and recipes developed specifically for the purpose of this research during a pilot study with the support of mothers from the local community. Portion sizes were estimated using a photographic atlas of foods commonly consumed by Ivorian infants, specifically developed for the study and appropriate for the age categories and by using household measures (glasses, cups, spoons, bowls, etc.). For breastmilk, quantities were estimated to be 95 and 150 mL per feed for 6–11 and 12–36 mo old, respectively, and a maximal quantity of 1120 and 810 mL per day (quantities of breastmilk could not exceed this amount regardless of the number of additional feeds) [[19], [20], [21], [22]]. For children aged 6–12 mo, an average breastmilk intake per feed was estimated around 95 mL because breastmilk daily intake has been estimated to be ∼760 mL [19,20] with an estimated number of feeds of 8 times per day. For children aged 12 mo and older, an average breastmilk intake per feed was estimated to be ∼150 mL because breastmilk daily intake has been estimated to be ∼600 mL [19,20] with an estimated number of feeds of 4 times per day. In the absence of precise data on maximal breastmilk intake beyond 12 mo, maximal intake was set a priori at 1120 and 810 mL for 6–12 and 12 and above months, respectively, from existing data showing maximal breastmilk intake ranging from 1009 g between 6 and 12 mo [21], 1144 g at 6 mo [20], 1185 g at 9 mo [20], 1154 g at 12 mo [20], 620 g (estimated) at 12 mo [22], 452 mL (estimated) at 18 mo [22], and 267 mL (estimated) at 24 mo [22], corresponding to 11–12 feeds per day on average for children aged 6–12 mo and 5–6 feeds for children aged 12 mo and older. To ensure qualitative dietary data collection, supervisors reviewed all dietary records at the end of each study day at each recruiting site. Any unclear information was immediately verified with the interviewer. Data were then entered into Excel by the supervisor and checked by 2 interns separately, and subsequently by the principal investigator, before being cleaned and then analyzed in R Studio.

### Assessment of dietary and energy intakes from the 24-h dietary intake questionnaire

Dietary intake (in g/day or mL/day) was assessed after classification of the consumed foods and beverages into 14 groups and additional subgroups. This classification was based on the harmonized and international groups and subgroups defined by the FAO/WHO, adjusted to reflect Ivorian food specificities.

Macronutrient and micronutrient intake was then evaluated using relevant food composition databases, such as the FAO Food Composition Table for Western Africa [23] for 64 of 84 items, the food composition table for use in the Gambia [24], and the Kenya food composition table [25] to complete the nutritional composition of specific food items, and the French food composition table [26] mainly for imported products, such as apple compote, chocolate spread or salami, or other sources for a few food items. The nutritional composition of 1 specific local food (Anagobaka) was extracted from a previous scientific publication [27]. Six local sauces were created directly from the recipe, using the ingredients and their weight, and the nutritional composition was calculated from the final recipe with a correcting factor for evaporation. Finally, for commercial infant products such as infant formula or fortified infant cereal, the nutritional composition was directly extracted from the label.

Dietary diversity was evaluated using 2 indices: the MDD and the dietary diversity score (DDS). The MDD can be used to monitor and assess the dietary quality of infants and young children and is therefore appropriate for identifying high-need populations, setting national-level targets, and monitoring and assessing interventions [28]. The MDD was calculated from the 24-h dietary intake questionnaire as the percentage of children who consume foods and beverages from ≥5 of 8 of the following 8 food groups within the previous day: *1*) breastmilk; *2*) grains, roots, tubers, and plantains; *3*) pulses (beans, peas, and lentils), nuts, and seeds; *4*) dairy products (milk, infant formula, yogurt, and cheese); *5*) flesh foods (meat, fish, poultry, and organ meats); *6*) eggs; *7*) vitamin A–rich fruits and vegetables; and *8*) other fruits and vegetables [28]. The outcome variable is coded as 1 if the children consumed ≥5 food categories in the last 24 h and therefore are considered to have adequate MDD, or coded as 0 if the children consumed <5 food categories in the last 24 h and are therefore considered to have inadequate MDD. The MDD is positively associated with the mean micronutrient adequacy of the diet [29]. The previous version of the MDD indicator guidance measured ≤7 food groups and did not capture breastmilk as a food source, thereby “penalizing” breastfed children in comparison to formula-fed children in assessing their diet quality. The 2021 guidance includes breast milk as 1 of the 8 food groups, which makes the comparison of MDD for breastfed and nonbreastfed infants more accurate [28].

A 10-point food group DDS was used by summing the number of food groups consumed from the 24-h dietary intake questionnaire. The 10 predefined food groups, underlying the DDS indicator, are as follows [30]: *1*) starchy staple foods; *2*) pulses (beans, peas, and lentils); *3*) nuts and seeds; *4*) dairy products (milk, yogurt, and cheese); *5*) flesh foods (meat, fish and seafood, poultry, and liver or organ meats); *6*) eggs; *7*) dark green leafy vegetables; *8*) vitamin A–rich fruits and vegetables; *9*) other vegetables; and *10*) other fruits. Individual scores are meant to reflect the nutritional quality of the diet. The DDS reflects the probability of micronutrient adequacy of the diet, and therefore, food groups included in the score are tailored toward this purpose. Previous research has shown that the fats and oils food group does not contribute to the micronutrient density of the diet, and this food group is part of neither the MDD nor the DDS.

Alongside the diet diversity assessment, particular attention was given to egg and/or flesh food (EFF) consumption. Indeed, WHO guiding principles for feeding breastfed and nonbreastfed children state that “meat, poultry, fish, or eggs should be eaten daily, or as often as possible,” because their regular consumption is associated with higher intakes of various nutrients important for optimal linear growth. The indicator to evaluate EFF intake was the percentage of children who consumed EFF during the previous day [28].

### Statistical analyses

Data on food consumption and the contribution of food groups to daily portions and energy intake were expressed as means. Comparisons between participants grouped by age, and digital status was performed using Student *t* tests, *χ*^2^ tests, and ANOVA, as appropriate. All statistical analyses were performed using the R software (version 3.6.1). The limit for statistical significance was set at *P* < 0.05.

## Results

### Study population

**FIGURE 1:**
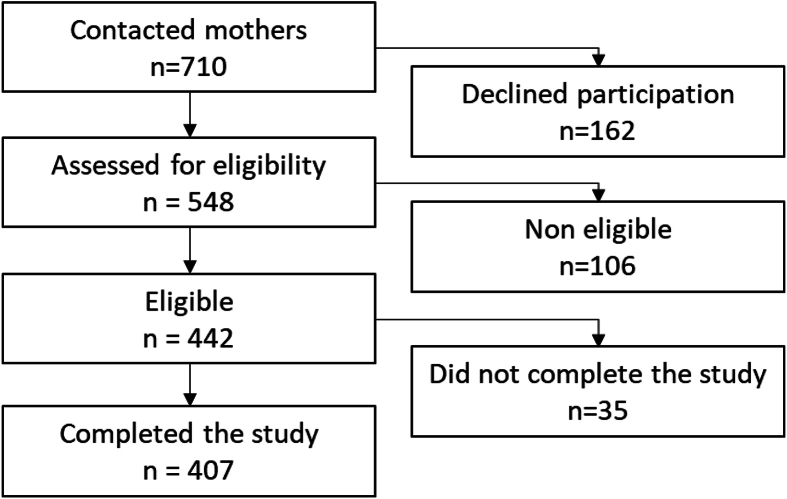
Flow chart of the study participants.

There were 407 children included in the study: 213 children aged 6–11 mo, 135 aged 12–23 mo, and 59 aged 24–36 mo. The first participant was recruited on August 11, 2022. The flowchart of the study is presented in Figure 1. The characteristics of the sample population are provided in Table 2. The sample population was 53% female. Most of the children were of normal ponderal status (62%); however, more children were overweight in the 24- to 36-mo-old group (43%) than in the 6–11 (12%) and 12- to 23-mo-old (27%) groups. The majority of children (95%) visited the health center for a routine visit, for their vaccination, to be weighed or to accompany their sibling for a visit and therefore did not have any health issue. Children from the younger age group mostly came for vaccination or to be weighed, whereas older children from the older group came mainly for routine visit or to accompany their sibling. Regarding their medical history, more than a third of the children from the study population had already contracted malaria, especially in the older age group (59% of 24- to 36-mo old, *P* < 0.0001 vs. the other groups). During the first 6 mo following birth, children from the study were mostly breastfed, either exclusively (37%), with the addition of water (11%) or with the addition of formula (47%), whereas 5% received formula only. Food diversification began at 5 mo or earlier for 14%, at 6 mo for 77% and at 7 mo, or later for 9% of children. As expected, mothers from the digital group (*n* = 107, 26%) were more educated (*P* < 0.0001) and more employed in the private or public sector (*P* < 0.0001) than mothers in the nondigital group (Table 3). The occupation of the father also differed between the digital and nondigital groups (*P* < 0.0001) with fathers from the digital mothers’ group working more as craftsmen or liberal activities and fathers from the nondigital mothers’ group working more as farmers or traders. The mean household size was 5.3, and the mean number of children younger than the age of 5 y in the household was 1.4 with no difference between homes from digital and nondigital mothers for neither household size nor the number of children younger than 5 y.

**TABLE 2:** Study population description by age category.

	Whole population (*n* = 407) 100%		6–11 mo (*n* = 215) 52%		12–23 mo (*n* = 134) 33%		24–36 Mo (*n* = 58) 15%		*P* value^1^
	Mean or *n*	SD or %	Mean or *n*	SD or %	Mean or *n*	SD or %	Mean or *n*	SD or %	
Age (mo)	14.2	7.5	8.6	1,6	16.7	3.0	29.1	3.8	<0.0001
Weight (kg)	9.5	2.2	8.3	1,4	10.2	2.0	12.4	1.7	<0.0001
Height (cm)	74.4	7.9	71.3	6,2	76.2	6.9	81.3	9.4	<0.0001
BMI (kg/m^2^)	17.3	4.2	16.5	3,5	17.8	4.2	19.4	5.4	<0.0001
Ponderal status 2^,^^3^	383		201		124		58		<0.0001
Underweight	61	16%	45	22%	12	10%	4	7%	
Normal	238	62%	131	65%	78	63%	29	50%	
Overweight	84	22%	25	12%	34	27%	25	43%	
Sex									
Females	216	53%	118	55%	71	53%	27	47%	0.53
Males	191	47%	97	45%	63	47%	31	53%	
Recruitment site									
INSP	134	33%	72	33%	44	33%	18	31%	0.98
Plateau hospital	140	34%	72	33%	46	34%	22	38%	
Pediatrics (Angré)	133	33%	71	33%	44	33%	18	31%	
Purpose of the visit									
Vaccination	167	41%	125	58%	37	28%	5	9%	<0.0001
Routine visit	100	25%	38	18%	40	30%	22	38%	0.001
Weighing	69	17%	42	20%	22	16%	5	9%	0.14
Accompanying sibling to visit	52	13%	14	7%	23	17%	15	26%	0.21
Other	31	8%	14	7%	9	7%	8	14%	0.16
Cold	17	4%	6	3%	9	7%	2	3%	0.20
Fever	12	3%	3	1%	7	5%	2	3%	0.12
Refuse to feed	5	1%	2	1%	2	1%	1	2%	0.84
Diarrhea	2	0%	1	0%	0	0%	1	2%	0.29
Pregnancy term	402^2^								
On term	367	91%	193	92%	121	90%	53	91%	0.87
Preterm	35	9%	17	8%	13	10%	5	9%	
Medical history of the child									
Sickle-cell anemia	1	0%	1	0%	0	0%	0	0%	-
Malaria	149	37%	58	27%	57	43%	34	59%	<0.0001
β-Thalassemia	0	0%	0	0%	0	0%	0	0%	-
Hemophilia	0	0%	0	0%	0	0%	0	0%	-
Other	194	48%	104	48%	68	51%	22	38%	0.25
None	121	30%	73	34%	35	26%	13	22%	0.13
Feeding practices between 0 and 6 mo									0.17
Mixed feeding (breastmilk and formula)	191	47%	96	45%	61	46%	34	59%	
Exclusive breastfeeding	151	37%	76	35%	56	42%	19	33%	
Predominant breastfeeding (breastmilk and water)	43	11%	30	14%	10	7%	3	5%	
Formula feeding	22	5%	13	6%	7	5%	2	3%	
Digital status of mothers									<0.001
Digitally-fluent mothers	107	26%	40	19%	47	35%	20	34%	
Non digitally-fluent mothers	300	74%	175	81%	87	65%	36	66%	

Abbreviations: *n*, number of children; SD, standard deviation.

1 *P* values for between-group comparisons were obtained using the *t* test and *χ*^2^ tests as appropriate.

2 n = XX total number of participants taking into account missing values for this variable.

3 For ponderal status, a corrected age was applied for premature children (born before 37 wk).

**TABLE 3:** Mother’s and household’s characteristics.

	Whole population (*n* = 407)		Digitally-fluent mothers (*n* = 107)		Non digitally-fluent mothers (*n* = 300)		*P* value^1^
	Mean or *n*	SD or %	Mean or n	SD or %	Mean or *n*	SD or %	
Age (y)	30.1	6.3	31.4	5.7	29.6	6.4	0.008
Education level							<0.0001
None	90	22%	3	3%	87	29%	
Primary school	57	14%	1	1%	56	19%	
Secondary school	107	26%	27	25%	80	27%	
Superior education	151	37%	76	71%	75	25%	
Unknown	2	0%	0	0%	2	1%	
Occupation							<0.0001
At home	143	35%	19	18%	124	41%	
Employed (private or public sector)	116	29%	56	52%	60	20%	
Trader	63	15%	7	7%	56	19%	
Craftswoman / liberal activity	49	12%	10	9%	39	13%	
Student	36	9%	15	14%	21	7%	
Marital status							0.76
Single	61	15%	17	16%	44	15%	
In a relationship	346	85%	90	84%	256	85%	
Occupation of the father							<0.0001
Unemployed	2	0%	2	2%	0	0%	
Employed (private or public sector)	7	2%	2	2%	5	2%	
Trader	91	22%	10	9%	81	27%	
Craftsman/liberal activity	200	49%	72	67%	128	43%	
Farmer	104	26%	20	19%	84	28%	
Does not know	3	1%	1	1%	2	1%	
Household size	5.4	2.4	5.3	2.3	5.6	2.4	0.39
Number of children under 5 in household	143	0.70	1.38	0.61	1.45	0.68	0.38

Abbreviations: *n*, number of children; SD, standard deviation.

1 *P* values for between-group comparisons were obtained using the *t* test and *χ*^2^ tests as appropriate.

### Dietary habits

Results from the baseline questionnaire were analyzed. Breastfeeding practices decreased with age from 85% of children in the 6–11 mo, 38% in the 12–23 mo, to 3% in the 24–36 mo-old groups with 58% (*n* = 236) of the total sample being breastfed (*P* < 0.0001) (Table 4). Breastfed children received on average 7–8 feeds per day regardless of age (*P* = 0.35), whereas the number of feeds from milk other than breastmilk decreased with age from 2.8 ± 1.4 in the 6–11, 0.5 ± 0.7 in the 12–23 to 0 ± 0 in the 24–36-mo-old groups (*P* = 0.04). One-third of children (*n* = 144, 35%) consumed family meals (solid food only), whereas the other two-thirds (*n* = 257, 63%) received complementary feeding (breastmilk or formula and solid food). Of the children receiving complementary feeding, 177 of 198 (89%) were breastfed in the 6–11-mo-old group, 51 of 57 (89%) in the 12–23-mo-old group, and 2 of the 2 (100%) in the 24–36-mo-old group. Complementary feeding decreased with age (from 92% of 6–11, 43% of 12–23, to 3% of 24–36 mo old), whereas family meals practices increased with age (from 5% of 6–11, 57% of 12–23, to 97% of 24–36 mo old). The average number of meals per day was 3.4 and increased with age from 2.9 in the 6–11, to 3.8 in the 12–23, and 4.3 in the 24–36-mo-old groups, respectively (*P* < 0.0001).

**TABLE 4:** Description of child feeding practices by age category.

	Whole population (*n* = 407) 100%		6–11 mo (*n* = 215) 52%		12–23 mo (*n* = 134) 33%		24–36 mo (*n* = 58) 15%		*P* value^1^
	Mean or *n*	SD or %	Mean or *n*	SD or %	Mean or *n*	SD or %	Mean or *n*	SD or %	
Feeding practices									<0.0001
Exclusive breastfeeding	3	1%	3	1%	0	0%	0	0%	
Mixed feeding (breastmilk + formula)	3	1%	3	1%	0	0%	0	0%	
Complement feeding (breastmilk or formula and solid food)	257	63%	198	92%	57	43%	2	3%	
Family meals (no breastmilk or formula)	144	35%	11	5%	77	57%	56	97%	
Number of meals per day	3.4	1.3	2.9	1.2	3.8	1.1	4.3	1.1	<0.0001
Children breastfed	236	58%	183	85%	51	38%	2	3%	<0.0001
Number of breastfeeds per day for breastfed children	7.8	3.4	7.9	3.3	7.2	3.7	7.0	7.1	0.35
Number of milk intake (other than breastmilk)	2.5	1.5	2.8	1.4	0.5	0.7	0	0	0.04

Abbreviations: *n*, number of children; SD, standard deviation.

1 *P* values for between-group comparisons were obtained using the *t* test and *χ*^2^ tests as appropriate.

### Distribution of daily food and energy intake from the 24-h dietary intake questionnaire

Mean daily food intakes were 1051 ± 340 g, 1027 ± 517 g, and 769 ± 414 g for children aged 6–11 mo, 12–23 mo, and 24–36 mo, respectively (*P* ≤ 0.0001) and 1003 ± 452 g for the overall study population.

**FIGURE 2:**
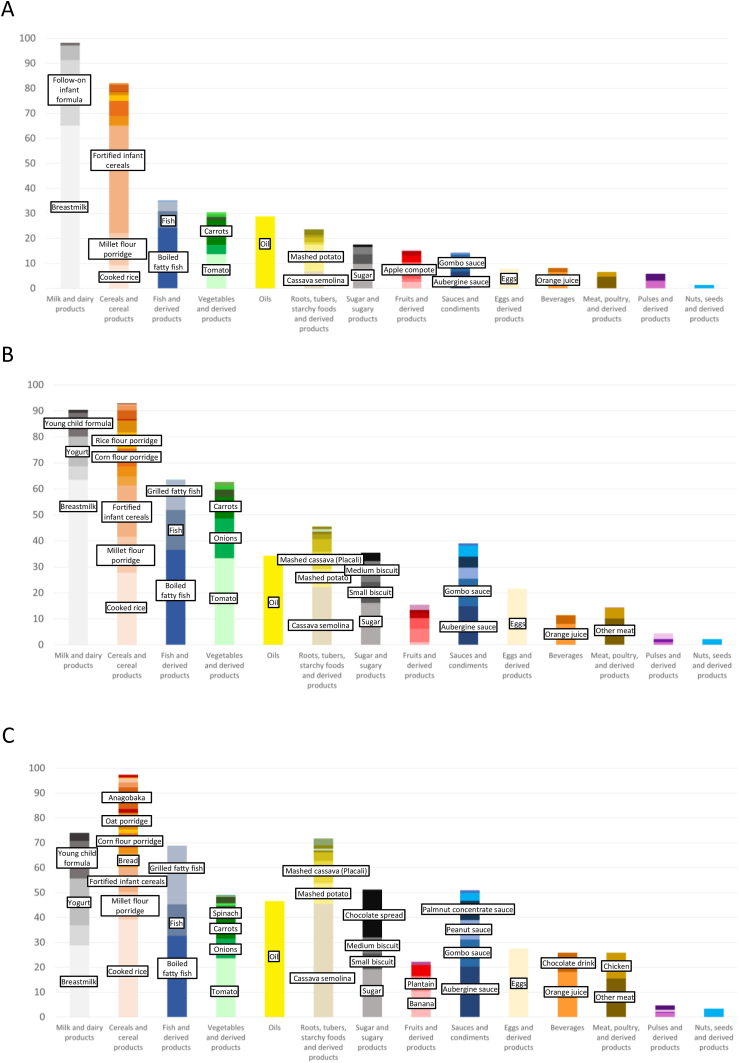

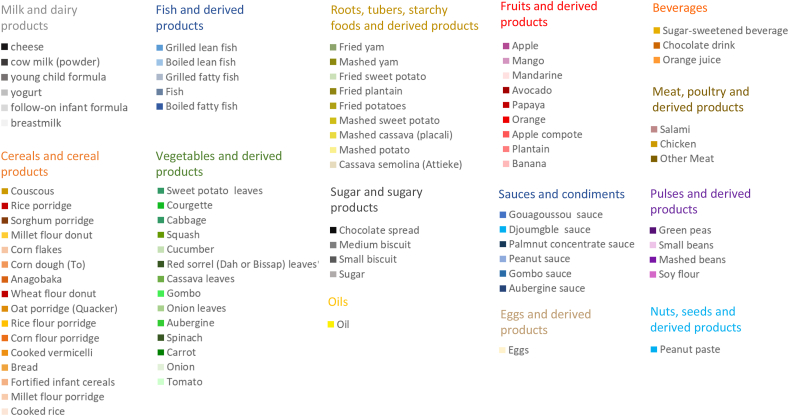
Evolution of the composition of the children’s diet from (A) 6–11 mo, (B) 12–23 mo until (C) 24–36 mo. The Y axis represents the percentage of consumers of the food category (e.g., 98.1% of children aged 6–11 mo consumed milk and dairy products). The colors within each food category represent the relative contribution of each food to the food category (e.g., breastmilk mean intake was 426 g/24 h, representing 66.2% of the “milk and dairy products” intake).

The most commonly consumed foods were milk (92% of study population, mainly breastmilk), cereal products (88% of study population, mainly rice, fortified infant cereals, mill flour or corn flour porridge), fish (49% of study population), vegetables (41% of study population, mainly tomatoes, carrots, and onions), root, tubers, and starchy foods [38% of study population, mainly cassava semolina (attieke), mashed potato, and mashed cassava (placali)], oil (33% of study population), sauces (26% of study population, mainly aubergine sauce, okra sauce, palmnut concentrate sauce, and peanut sauce), sugar (23% of study population, mainly table sugar, biscuits, and chocolate spread), whereas meat was only consumed by 11% of the total study population, fresh fruits by 9%, eggs by 15%, and pulses by 3% (Supplemental Table 1 and Figure 2).

The largest contributors to the portion in the overall study population was milk and dairy products (contributing to 64% of the portion), followed by cereals and cereal products (21%), roots, tubers, starchy foods, and derived products (4%), and vegetables (3%). In terms of food subcategories, milk contributed to 58% of the total portion; followed by cereal products (16%), dairy products (6%), cereals (5%), root vegetables (3%), vegetables (3%), tubers (2%), and fish (2%) (Figure 2). Although the contribution of milk and dairy products to the portion significantly decreased with age [74%, 59%, and 29% for children aged 6–11 mo, 12–23 mo, and 24–36 mo, respectively (< 0.0001)], that of cereals and cereal products [17%, 24%, and 32% for children aged 6–11 mo, 12–23 mo, and 24–36 mo, respectively (*P* = 0.0004)], roots, tubers, starchy foods, and derived products [2%, 5%, and 14% for children aged 6–11 mo, 12–23 mo, and 24–36 mo, respectively (*P* < 0.0001)], and to a lesser extent fish and derived products, meat, poultry and derived products, eggs and derived products, sauces, and vegetables and derived products significantly increased with age. Despite their nutritional interest, the consumption of pulses and derived products and nuts, seeds, and derived products remained marginal both in terms of quantity (contribution to 0.3% of the food portion) and number of consumers (*n* = 12, 3%). Fortified infant cereals were consumed by 149 children (37%), with a larger proportion of younger children consuming fortified infant cereals compared with the older age groups [48%, 28%, and 12% for children aged 6–11 mo, 12–23 mo, and 24–36 mo, respectively (*P* < 0.0001)].

Mean daily energy intakes were 867 ± 329 kcal, 1001 ± 409 kcal, and 906 ± 365 kcal for children aged 6–11 mo, 12–23 mo, and 24–36 mo, respectively (*P* = 0.004) (Supplemental Table 1) and 917 ± 366 kcal for the overall study population. The largest contributor to energy intake in the overall study population was milk and dairy products (49%), followed by cereals and cereal products (29%), roots, tubers, starchy foods and derived products (7%), and fish and derived products (4%) (Figure 2). In terms of food subcategories, milk contributed to 44% of energy intake; followed by cereal products (23%), cereals (6%), dairy products (5%), root vegetables (5%), fish (4%), tubers (3%), and oil (2%). Although the contribution of milk and dairy products to energy intake significantly decreased with age [63%, 43%, and 18% for children aged 6–11 mo, 12–23 mo, and 24–36 mo, respectively (*P* < 0.0001)], that of cereals and cereal products [25%, 32%, and 37% for children aged 6–11 mo, 12–23 mo, and 24–36 mo, respectively (*P* < 0.0001)], roots, tubers, starchy foods, and derived products [4%, 7%, and 19% for children aged 6–11 mo, 12–23 mo, and 24–36 mo, respectively (*P* < 0.0001)], and to a lesser extent fish and derived products, meat, poultry and derived products, eggs and derived products, oils, sauces, sugar and sugary products, and vegetables and derived products significantly increased with age (Supplemental Table 1). Hence, the energy contribution of the milk and dairy product category, characterized by its high nutritional quality, was mainly replaced by cereals, cereal products and derived products, and roots, tubers, starchy foods and derived products, with limited nutritional value.

**FIGURE 3:**
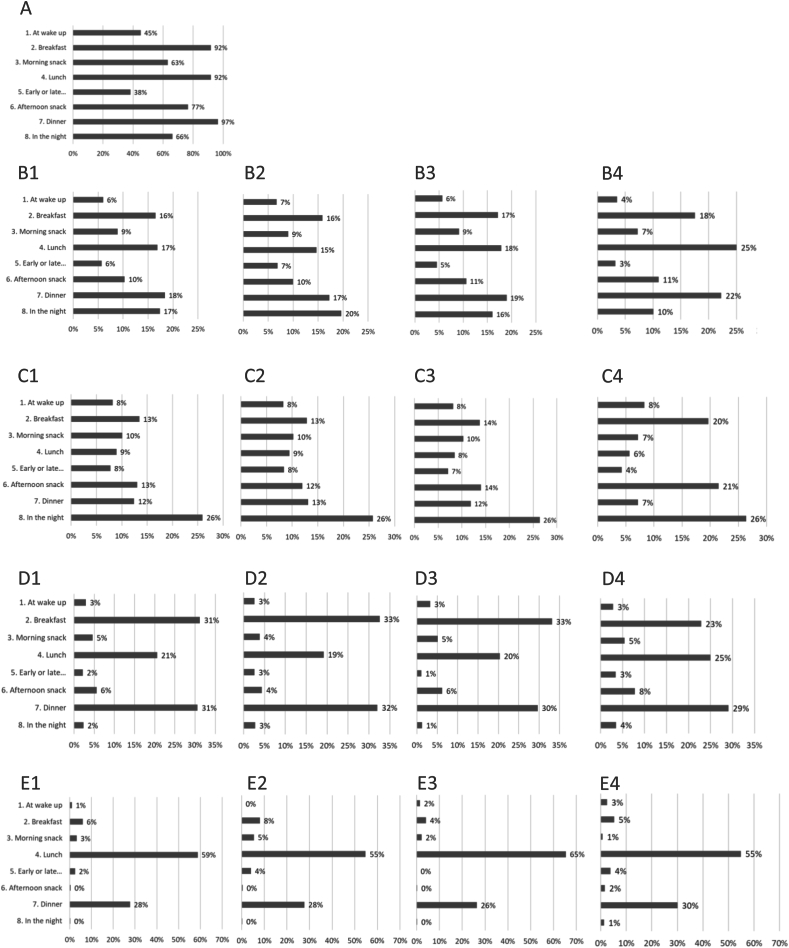
Description of the consumption according to the moments of consumption. (A) Percentage of children consuming each meal. (B) Repartition of portion throughout the day in B1 all children (6–36 mo) B2 6–11 mo-old children B3 12- to 23-mo-old children B4 24- to 36-mo-old children. (C) Repartition of milk and derived products portion throughout the day in C1 all children (636 mo) C2 6- to 11-mo-old children C3 12- to 23-mo-old children C4 24-to 36 mo-old children. (D) Repartition of cereals and derived products portion throughout the day in D1 all children (6–36 mo) D2 6- to 11-mo-old children D3 12- to 23-mo-old children D4 24- to 36-mo-old children. (E) Repartition of vegetables and derived products portion throughout the day in E1 all children (6–36 mo) E2 6- to 11-mo-old children E3 12- to 23-mo-old children E4 24- to 36-mo-old children.

Food and beverage consumption was centered around 3 main meals: breakfast, lunch, and dinner consumed by 92%, 92%, and 97% of children, respectively, and contributing to 16%, 17%, and 18% of the portion and 18%, 19%, and 20% of the energy intake, respectively. Interestingly, consumption during the night (66% of children) also contributed to 17% of the portion and 14% of energy intake. In terms of age difference, the food and beverage consumption of younger children aged 6–11 mo was more spread throughout the day, including the night, which contributed the most in terms of portion, compared with older children aged 24–36 mo for whom consumption was more concentrated around the 3 main meals (Figure 3). In terms of food repartition, milk and dairy products were consumed equally throughout all moments of the day, and more specifically at night, for younger children but mainly during breakfast, afternoon snack, and night for older children, whereas other food categories, such as cereal and derived products, roots, tubers, starchy foods and derived products, were consumed mainly during the 3 main meals (breakfast, lunch, and dinner), and vegetables, fish, and meat were consumed mainly at lunch and dinner regardless of age (Figure 3).

Breakfast was mainly composed of milk and derived products (52%) and cereals and derived products (39%), whereas lunch was composed of a variety of foods, including milk and derived products (34%); cereals and derived products (25%); roots, tubers, and derived products (14%); vegetables and derived products (11%); fish and derived products (8%); and sauces (3%). Dinner was also composed of a variety of foods but with a larger contribution of milk and cereals (milk and derived products 43%; cereals and derived products 35%; roots, tubers, and derived products 6%; vegetables and derived products 5%; and fish and derived products 4%). Morning (*n* = 257 children, 63%) and afternoon (*n* = 312 children, 77%) snacks were mainly composed of milk and derived products (73% including 63% milk for the morning snack and 81% including 46% milk for the afternoon snack) and cereals and derived products (11% for both morning and afternoon snacks). The night intake (*n* = 270 children, 66%) was almost entirely characterized by milk intake (94%).

### Diet diversity

The DDS, as measured by the quantitative number of food categories from which foods were consumed within a predefined list, was calculated for each participant as a mean to determine variety in the diet. The mean DDS for the overall study population was 3.3 ± 1.4 (Table 5). The DDS increased with age from 2.9 ± 1.4 for 6–11-mo-old children to 3.9 ± 1.0 for 24- to 36-mo-old children (*P* < 0.0001) meaning that children consumed foods from more diverse food categories as they aged. There was no significant difference in the DDS between children from digital mothers (3.5 ± 1.5) and from nondigital mothers (3.2 ± 1.4) (Table 6).

**TABLE 5:** Prevalence of children meeting the minimum dietary diversity score in each age category and in the overall study population.

	Whole population (*n* = 407) 100%		6–11 mo (*n* = 215) 52%		12–23 mo (*n* = 134) 33%		24–36 mo (*n* = 58) 15%		*P* value
	Mean or *n*	SD or %	Mean or *n*	SD or %	Mean or *n*	SD or %	Mean or *n*	SD or %	
DSS	3.3	1.4	2.9	1.4	3.6	1.4	3.9	1.0	<0.0001
Meeting the MDDS	107	26%	45	21%	46	34%	16	28%	0.02
Not meeting the MDDS	300	74%	170	79%	88	66%	42	72%	

Abbreviation: MDDS, minimum dietary diversity score.

**TABLE 6:** Prevalence of children meeting the minimum dietary diversity score in digital or nondigital mothers’ groups.

	Whole population (*n* = 407) 100 %		Children from digitally-fluent mothers (*n* = 107) %		Children from non digitally-fluent mothers (*n* = 300) %		*P* value
	Mean or *n*	SD or %	Mean or *n*	SD or %	Mean or *n*	SD or %	
DDS	3.3	1.4	3.5	1.5	3.2	1.4	0.13
Meeting the MDDS	107	26%	30	28%	77	26%	0.63
Not meeting the MDDS	300	74%	77	72%	223	74%	

Abbreviation: MDDS, minimum dietary diversity score.

Three quarters (74%) of children from the study population had inadequate diet diversity as evaluated by the minimum diet diversity score (MDDS), which reports the prevalence of children consuming foods from 5 or more different food categories (Table 5). As expected, the percentage of children meeting the MDDS was the lowest in the younger 6- to 11-mo-old group (21%). However, although the percentage of children meeting the MDDS increased in the 12- to 23-mo-old group (34%), it was lower in the 24- to 36-mo-old group (28%) (*P* = 0.02). The prevalence of children not meeting the MDDS was similar in the digital mothers’ group (72%) and in the nondigital mothers’ group (74%) (Table 6).

Although dietary diversity was relatively low for the overall study population (mean DDS <4), when dividing children into DDS terciles, it was possible to observe differences in food category consumption (Table 7). All children with low, medium, or high relative dietary diversity consumed similar staple foods consisting of dairy products and starchy staple food. However, the diet of children with medium relative dietary diversity was also characterized by the consumption of flesh foods (fish and meat) in addition to dairy products and starchy foods, and that of children with high relative dietary diversity also contained vitamin A–rich fruits and vegetables as well as other vegetables in addition to flesh foods, subsequently driving higher nutrient adequacy.

**TABLE 7:** Most commonly consumed food categories in children with low, medium, and high relative dietary diversity.

	Whole population (*n* = 407) 100%		1st tertile of DDS (*n* = 139) %		2nd tertile of DDS (*n* = 178) %		3rd tertile of DDS (*n* = 90) %	
	Mean	SD	Mean	SD	Mean	SD	Mean	SD
DDS	3.3	1.4	1.7	0.4	3.5	0.5	5.3	0.5
Food groups consumed by 50% at least of children	Dairy products, starchy staple foods, flesh foods		Dairy products, starchy staple foods		Dairy products, starchy staple foods, Flesh foods		Dairy products, Other vegetables, starchy staple foods, Flesh foods, Vitamin A–rich fruits and vegetables	

Abbreviation: DDS, dietary diversity score.

Looking at the consumption of EFF specifically showed that 60% of children in the study had consumed eggs or flesh food the previous day. This was mainly driven by fish consumption, which was relatively high compared with meat and egg consumption. Within the 6- to 23-mo-old children, this percentage was much lower, reaching only 46%.

## Discussion

This study adds significant and recent knowledge about the feeding practices of Ivorian mothers and eating behaviors of 6- to 36-mo-old healthy children in Côte d’Ivoire, bringing additional supporting data for nutritional recommendations and policies.

Regarding breastfeeding practices, 95% of children from the study received breastmilk in the first 6 mo of their lives. However, only 37% were exclusively breastfed during the entire first 6-mo period, as per WHO and national recommendations [14]. This is slightly higher than the exclusive breastfeeding rates recorded at 34% in the 2021 National Demographic and Health Survey [31] and much higher than that previously recorded rates from 2016 standing at 23.1% [32]. It is, however, much below the objective that the Ivorian government set in its National Strategic Plan for Mother, Newborn, and Child Health, to reach a rate of 50% by 2020 [33] and far from the 72% target by 2025. Interestingly, exclusive breastfeeding rate was higher for male infants (41%), than for female infants (33%), as previously observed in a different sample of both rural and urban populations [34]. Exclusive breastfeeding rates were identical in children from digitally fluent (37%) and nondigitally fluent mothers (37%), despite the difference in education between the 2 groups. Previously published data had shown that mothers with either no education or primary education were more likely to breastfeed, although this was moderated by the child’s health status [34].

For a large majority of children (77%), food diversification began at 6 mo as per the WHO and national recommendations. Breastfeeding rates decreased with age, as expected. Although nonexclusive breastfeeding was continued for 85% of children in the 6- to 11-mo-old group, only 38% of children in the 12- to 23-mo-old and 3% of children in the 24- to 36-mo-old group were breastfed. In a recently published study by Janmohamed et al. [35] involving 118 children aged 6–23 mo from rural Côte d’Ivoire, breastfeeding rates were 96% in the 6- to 11-mo-old group and 65% in the 12- to 23-mo-old group. This difference may be due to the selection of the sample, because breastfeeding rates may be higher in rural areas and poorer socioeconomic households. The WHO recommends exclusive breastfeeding for the first 6 mo of life for optimal growth, development, and health and breastfeeding continuation alongside food diversification up until 2 y of age or beyond. Breastmilk remains an important source of energy and nutrients for children aged between 6 and 23 mo, which may cover half or more and a third or more of energy needs of 6- to 12-mo-old and 12- to 24-mo-old children, respectively. Breastmilk also represents an essential source of energy and nutrients in case of illness. In sick children who have little or no appetite for solid food, breastfeeding can help prevent dehydration and provide the nutrients required for recovery and reduced mortality rates in malnourished children. Prolonged breastfeeding also decreases risk of breast and ovarian cancer in mothers and can prevent closely spaced pregnancies, which may cause a risk of malnutrition for mothers [36]. Beyond its positive effect on nutritional status, continued breastfeeding has been consistently associated with higher performance in intelligence tests among children and adolescents, with those breastfed longer than 12 mo benefiting the most.

Complement feeding (i.e., breastmilk or formula, complemented with solid food) rates decreased from the 6- to 11-mo-old to the 24- to 36-mo-old groups with only 3% of children in the older group received milk in complement to solid food, whereas family meals (solid food only) increased with age from 5% in the 6- to 11-mo-old to 97% in the 24- to 36-mo-old groups. Although the number of breastmilk intakes per day remained the same in breastfed children across the different age categories, the number of meals consumed per day increased with age from 2.9 meals per day for the 6- to 11-mo-old age category to 3.8 meals and 4.3 per day for the 12- to 23-mo-old and 24- to 36-mo-old age categories, respectively. However, it remained significantly under the global recommendations from the WHO, i.e., to consume 3–4 meals with 1 or 2 snacks between 9 and 23 mo to ensure diet diversity and energy and nutrient adequacy [36], and under the Côte d’Ivoire National Nutrition Directives from 2017, which recommend that children aged 6–9 mo, 9–24 mo, and 24–59 mo consume 2–3 meals with the addition of 1–2 snacks, 3–4 meals with the addition of 1–2 snacks, and 3–4 meals with the addition of 2 snacks, respectively [18].

In terms of food consumption, foods that were the most commonly consumed and contributed the most to the portion and the energy intake were milk; cereals such as rice, mill flour, or corn flour porridge; roots, tubers, and starchy foods; fish, vegetables, oils; and sauces. Interestingly, only 37% of children consumed fortified infant cereals overall, and only 12% in the 24- to 36-mo-old age category, for which breastmilk, formula milk, meat, and egg consumption were also relatively low. The consumption of many foods with high nutrient density, such as meat, fresh fruits, eggs, pulses, and seeds and nuts was relatively low, with less than a quarter of children from all different age categories consuming them. Although there are limited data on food consumption and feeding practices of preschool children in Côte d’Ivoire, an analysis of the Demographic and Health Survey data sets of 33 countries in sub-Sahara showed that, similar to our results, only 13%, 19%, and 37% of children aged 6–23 mo consumed eggs, fruits (other than vitamin-rich fruits), and flesh food, respectively [37]. The recent study by Janmohamed et al. [35], looking at the intake of different food categories in children aged 6–23 mo in rural Côte d’Ivoire, demonstrated higher intakes of eggs (19.5%); flesh foods (52.5%); and pulses, nuts, and seeds (53.4%) in this population than in our study group. This may be explained by the different geographical area, the rural population selected (although previous studies have shown that rural residence is associated with lower dietary diversity) [37], and different study participant selection. With increasing age, the consumption of milk and dairy products and their contribution to portion and energy intake decreased quite drastically, leaving room for the most common staple foods such as cereals, roots, tubers, and starchy foods, and to a lesser extent to animal protein sources, such as fish and meat, and accompanying vegetables and sauces. The low consumption of nutrient-dense foods, such as meat, eggs, fruits, offal, pulses, legumes, and nuts and seeds, may be explained by unaffordability, lack of consumption habit, or beliefs and social taboos. For example, the relatively high consumption of fish compared with meat and egg consumption may be explained by differences in cost but not availability because all products are available in the country. In the Abidjan area, the main fish used by the mothers for their infants are tuna, mackerel, and Sosso (common Atlantic bass or small head bass). One kilogram of tuna costs ∼1500 CFA, 1 kg of mackerel costs ∼1500 CFA, and 1 kg of Sosso fish costs ∼2500 CFA. In comparison, a kilogram of meat with bones costs ∼3500 CFA and 30 eggs ∼2500 CFA. Fish is regarded as a cheaper yet nutrient-rich source of protein for infants in Côte d’Ivoire. Fish is also recommended by health workers to mothers of young infants because it is nutrient rich and believed to be easily digested by infants younger than 1 y. Mothers who are used to giving their children fish continue with this habit after the child turns 1 y. The consumption of eggs is low in children. This may be explained by social taboos around the consumption of eggs. In Côte d’Ivoire, many mothers avoid giving eggs to their infants because of the culturally held belief that eating eggs causes children to either become thieves later in life—a taboo also documented in other West African feeding practices more broadly [38,39]—or be bloated [40]. In a previous study carried out in the Abidjan area, eggs were shown to be among the most avoided foods because of social beliefs and taboos [40]. As for the low consumption of fruits, it may be explained by their cost, which varies greatly according to the season. The consumption of offal, pulses, and legumes is almost inexistent as mothers rarely know these foods. A recent local study from the National Institute of Public Health (Institut National de Santé Publique, INSP) also highlighted the low consumption of offal, eggs, and pulses (results not published). Results from both studies will be used to update training modules of the nutrition program ran by INSP with a specific focus on improving the awareness of these foods for mothers through education as a key element.

Meals were relatively equivalent in terms of contribution to the portion and energy intake of the day, but the study population was also characterized by significant nightly intake. The consumption of younger children was spread throughout the different meals of the day, including a significant nightly intake, as expected, whereas older children concentrated the majority of their intake around breakfast, lunch, and dinner, with a smaller contribution of morning and afternoon snacks. Cereals, roots, tubers, and starchy foods were consumed equally throughout the day. Milk was consumed at night for young children, and breakfast, afternoon snack, and night for older children specifically. Other components of traditional meals such as fish, vegetables, and sauces were mainly consumed at lunch and dinner, whereas morning and afternoon snacks were composed of milk and cereals.

Beyond food intake, dietary diversity is of specific importance for the nutritional status and health of children. The DDS, measured by a quantitative number of food groups consumed by children during a 24-h window, has become a widely used method of determining variety in the diet, and by proxy, nutrient adequacy. Multiple studies have found a positive association between DDS and nutritional status or nutrient adequacy [[41], [42], [43], [44]]. A low DDS has also been associated with low weight and stunted growth [9,45]. A study by Aboagye exploring the association between diet diversity and undernutrition in children aged 6–23 mo from sub-Saharan countries found that having adequate MDD was associated with 12% less likelihood of being stunted, 13% reduced odds of wasting among children, and significantly lowered risk of underweight by 17% compared with those who had inadequate MDD [9]. In our study, dietary diversity significantly increased with age, from 2.9 in the 6- to 11-mo-old age group to 3.9 in the 24- to 36-mo-old group, with a larger proportion of children in the older groups meeting the MDD score than in the younger age group. The overall proportion of children in the study population not meeting the MDD score was 74%. This is very close to the pooled magnitude of inadequate MDD intake among children aged 6–23 mo in sub-Saharan countries standing at 77% but much lower than the one calculated for Côte d’Ivoire in this analysis at 89% [37]. This could be because data used for this analysis dates from 2011, and since then, there has been an improvement in the nutritional situation in Côte d’Ivoire. It could also be due to differences in the study population, with our study concentrated on children from the Abidjan area. Indeed, the previous study showed that dietary diversity was lower in rural areas. Variety is often much greater in urban and periurban centers, where food markets are adequately supplied and easily accessible [46]. There were also methodological differences in the calculation of the MDD between the 2 studies because the categories used for the score were slightly different in the aforementioned study. In the study from Aboagye et al. [9], the overall prevalence of inadequate MDD was 75% across all countries, with a prevalence of 89% for Côte d’Ivoire. In a more recent study from Janmohamed et al. [35], dietary diversity was calculated in a sample of 118 children aged 6–23 mo. The prevalence of inadequate dietary diversity was much lower in this study, 46% overall, 63% in the 6- to 11-mo-old group (compared with 79% in our study), and 31% in the 12- to 23-mo-old group (compared with 66% in our study) in line with the higher observed intake of eggs, flesh foods, pulses and nuts, and vitamin A–rich fruits and vegetables [35]. Again, this may be explained by the difference in geographical selection of the study population, although the sample used in their study was exclusively from a rural area and demonstrated higher dietary diversity in opposition to previous findings. Diet also varies across seasons, but the effect of seasonality on the dietary diversity of young children has seldom been investigated in Côte d’Ivoire.

Looking at food dietary diversity tertiles, although 50% of children consumed dairy products and starchy staple foods in all dietary diversity groups, only in the group that had the highest DDS did 50% children consume flesh foods and vitamin A–rich fruits and vegetables, suggesting that fruits and meat consumption may be 2 key food categories to promote in young children in Côte d’Ivoire. Another dietary quality indicator was also used in the study: the prevalence of eggs and flesh food consumption in 6- to 23-mo-old children was 46% in our sample. The WHO highlights that there is evidence that children who consume eggs and flesh foods have higher intakes of various nutrients important for optimal linear growth, including energy, protein, essential fatty acids, vitamin B12, vitamin D, phosphorus, zinc, and selenium. Introduction of meat as an early complementary food for breastfed infants is associated with improved protein and zinc intake [28].

Previous studies have shown that maternal health care utilization status, sociodemographic factors such as maternal education, residence, mother’s occupation, economic factors, and media exposure of the mothers may be determinants of dietary diversity [9,47]. In our study, there was no significant difference in mean DDS between children of digitally fluent and nondigitally fluent mothers, nor was there a difference in the proportion of children meeting the MDD score between the 2 groups. Because the digital fluency of the mother was clearly associated with higher education as well as employment situation, it is unclear why we did not find a significant difference between the 2 groups. Although foods from all categories are available in the country, the low intake of specific categories such as eggs, pulses, nuts, and seeds is most likely because of lack of knowledge of these foods, lack of awareness of their importance, or the prevalence of social taboos and beliefs, which can override nutritional knowledge provided by health professionals [40]. Reinforcing nutritional education through long-term and persistent communication to mothers is key to changing feeding practices. It is also possible that our sample was not large enough to observe such differences or that other socioeconomic factors may play a role. Future analyses should focus on identifying the determinants of dietary diversity, including the socioeconomic factors in our study.

Individual DDSs are rapid and efficient means to estimate nutrient adequacy of the diet. Using a simplified 24-h recall tool such as the Iron Calculator could allow health professionals or digitally fluent caregivers to ascertain a child’s DDS over the previous 24 h. Combining a low DDS value with a low weight-for-age and weight-for-height value may be a useful way to identify children at a risk of undernutrition [46].

When interpreting the DDS, it is important to keep in mind that it does not indicate the quantity of food consumed. In our study, we used a semiquantitative 24-h recall, using a specifically developed food portion booklet with household measurements, such as bowls, cups, spoons, etc.

Our study provides new dietary intake and child feeding practices data in a population of young children aged 6–36 mo from Abidjan area in Côte d’Ivoire. It is often difficult to access recent and quantified dietary intake data in young children from Western African countries. First, beyond the usual difficulties of a 24-h recall including memory challenges and portion evaluation, collecting dietary intake data bears specific difficulties in young children: assessing ingested food quantities is challenging because portion sizes are specific to this population, food may be left unconsumed or thrown on the floor, children themselves cannot assess or express how much they consumed, and in a family setting, the main caregiver is not always the person feeding the child or attending the child during his/her meal. Furthermore, cooking recipes and foods may be distinctive of a specific region or culture, making the use of global food composition tables complicated. To overcome these challenges, we developed specifically for this study a list of commonly consumed food and recipes along with the associated food portion sizes with photos of containers and dishes appropriate to the age group with the support of mothers from the local community. We also used local food composition tables to calculate the nutritional composition of the foods. Assessing breastfeeding quantities bears its own difficulty because it is practically impossible to evaluate precise quantities without the use of before- and after-breastfeed weighing, which is burdensome and requires a trained researcher to follow the mother and her child for 24 h. We acknowledge that breastmilk quantities and its contribution to nutrient intake are only estimated, because we used average breastmilk quantities per feed estimated by previous research. The lack of data on maximal breastmilk intake beyond 12 mo was also a major point of uncertainty. Nevertheless, the implications of this uncertainty were minimal, given that only a small proportion of infants in our study continued breastfeeding beyond 12 mo. Finally, although our study population is not representative of the overall population in Côte d’Ivoire as we recruited children within the Abidjan region as well as selected healthy children only, we ensured that a large sample, with specific representation of different age groups, as well as mothers from different socioeconomic backgrounds, was recruited. It is important, however, to keep in mind that differences between our results and previously published results may arise from the selection of this population. The selection of children from different age categories allows for the observation of evolutions in eating and drinking habits of young children and the identification of specific populations of interest for targeted interventions and policies.

Future analysis will focus on adherence to the nutritional recommendations both for macronutrients and micronutrients, and for iron in particular, as well as identifying whether the Iron Calculator is a valuable tool in the identification of children with low iron intake.

In conclusion, our study in a sample of young children aged 6–36 mo from the Abidjan area in Côte d’Ivoire provides recent dietary intake and child feeding practices data. It highlights progress in some indicators such as the prevalence of exclusive breastfeeding in the first 6 mo of life, as well as some strengths in terms of dietary diversity, such as the early and common introduction of fish and fish products, and the continuation of breastfeeding past 6 mo of age. However, our results also showed that feeding practices and intake of fruits, meats, eggs, and pulses is suboptimal. This calls for interventions to improve child feeding practices in this age group through the renewal of nutritional recommendations and policies aiming to improve the availability and affordability of certain nutrient-dense food groups, such as fruits, meats, eggs, and pulses and the knowledge of mothers, particularly during the food diversification period. Intensifying nutritional education, especially for mothers of young children below 1 y old, is key to improving dietary diversity and nutrient-dense food intake in young children. Targeting this age group is important for 2 reasons: dietary diversity is very low between 6 mo and 1 y and feeding practices persist throughout childhood. Directing communication on affordable and available dietary sources of iron such as pulses, offal, and eggs, which today are seldom used by mothers, combining this education with teaching on cooking methods, and deconstructing deeply anchored beliefs and taboos would be most efficient. Micronutrient supplementation as well as food fortification are also essential elements of successful nutritional programs. Second, continuing the fight against infections and infestations, through the use of impregnated mosquito nets, systematic deworming of children is essential alongside nutritional education and supplementation. Increasing the intake of nutrient-dense foods is crucial for children to meet the MDD necessary to ensure nutritional adequacy and future health.

## Author contributions

The authors’ responsibilities were as follows – JHB, SPS, AC, PD-P, SOPA-T: designed research; SPS, AC: conducted research, with the support of SOPA-T; JHB, PD-P, JD: cleaned and analyzed the data; JHB, SPS: wrote the article; JHB: had primary responsibility for final content; and all authors contributed to interpretation of the study, read and approved the final manuscript. The authors would like to thank all the interviewers for their role in the data collection, and all participants who took part in the study.

## Data availability statement

Data described in the manuscript, code book, and analytic code will be made available upon request pending application and approval by the National Institute of Public Health, Abidjan, Côte d’Ivoire.

## Funding

Danone Nutricia Research funded the study. The supporting source had no restrictions regarding the publication of the results. Danone Nutricia Research provided the Institut National de Santé Publique, service de Nutrition, with Masimo devices and digital tablets for the purposes of the study.

## Conflict of interest

JHB, PD-P, and JD are employees of Danone Research. SOPA-T reports financial support, article publishing charges, equipment, drugs, or supplies, and statistical analysis were provided by Danone Research. SPS and AC have no conflicts of interest.
