# Supplementary material for: Feeding Practices Among Children Aged 6–36 Months Living in Urban Abidjan, Cote D’Ivoire: The Victory Cross-Sectional Study
Source: Curr Dev Nutr. 2025 Aug 26;9(10):107536. doi: 10.1016/j.cdnut.2025.107536 (PMC12509748; doi:10.1016/j.cdnut.2025.107536)
Supplement: Multimedia component 1 [file mmc1.docx]

**FEEDING PRACTICES AMONG CHILDREN AGED 6 TO 36 MONTHS LIVING IN URBAN ABIDJAN, COTE D’IVOIRE: THE VICTORY CROSS-SECTIONAL STUDY**

Jeanne H Bottin

**Supplementary Table 1:**

|  | **Whole population** | (n=407) |  |  | **6-11 months** | (n=215) |  |  | **12-23 months** | (n=134) |  |  | **24-36 months** | (n=58) |  |  | p-value | p-value |
| --- | --- | --- | --- | --- | --- | --- | --- | --- | --- | --- | --- | --- | --- | --- | --- | --- | --- | --- |
|  | Mean Intake in population (/24h) | Mean Intake in consumers (/24h) | Contribution to energy intake | % of consumers in population | Mean Intake in population (/24h) | Mean intake in consumers | Contribution to energy intake | % of consumers in population | Mean Intake in population (g/24h) | Mean Intake in consumers (/24h) | Contribution to energy intake | % of consumers in population | Mean Intake in population (g/24h) | Mean Intake in consumers (/24h) | Contribution to energy intake | % of consumers in population | Mean intake in consumers | %consumers |
| **All foods** | **1003** |  |  |  | **1051** |  |  |  | **1027** |  |  |  | **769** |  |  |  |  |  |
| **Milk and dairy products** | **643** | **698** | **49,3%** | **92,1%** | **781** | **796** | **62,6%** | **98,1%** | **605** | **670** | **43,1%** | **90,3%** | **221** | **298** | **17,8%** | **74,1%** | **<0.0001** | **<0.0001** |
| Breastmilk | 426 | 745 |  | 57,2% | 519 | 672 |  | 77,2% | 425 | 919 |  | 46,3% | 86 | 997 |  | 8,6% |  |  |
| Follow-on Infant formula | 125 | 347 |  | 36,1% | 209 | 366 |  | 57,2% | 35 | 211 |  | 16,4% | 24 | 697 |  | 3,5% |  |  |
| Yogurt | 58 | 140 |  | 41,0% | 46 | 129 |  | 35,3% | 77 | 152 |  | 50,8% | 56 | 142 |  | 39,7% |  |  |
| Young child formula | 30 | 229 |  | 13,0% | 7 | 204 |  | 3,3% | 61 | 231 |  | 26,1% | 45 | 236 |  | 19,0% |  |  |
| Cow milk (from powder) | 4 | 33 |  | 12,5% | 1 | 35 |  | 2,8% | 7 | 34 |  | 20,9% | 9 | 31 |  | 29,3% |  |  |
| Cheese | 0,3 | 14 |  | 2,2% | 0,1 | 10 |  | 0,9 % | 0,5 | 14 |  | 3,7% | 0,6 | 18 |  | 3,5% |  |  |
| **Cereals and cereal products** | **209** | **238** | **29,1%** | **88%** | **179** | **218** | **24,7%** | **82,3%** | **242** | **259** | **32,1%** | **93,3%** | **246** | **255** | **37,3%** | **96,6%** | **0.05** | **<0.0001** |
| Cooked rice | 48 | 129 |  | 37,6% | 20 | 98 |  | 20,5 % | 72 | 130 |  | 55,2% | 100 | 166 |  | 60,3% |  |  |
| Millet flour porridge | 31 | 144 |  | 21,4% | 28 | 137 |  | 20,5 % | 36 | 156 |  | 23,1% | 28 | 136 |  | 20,7% |  |  |
| Fortified infant cereals | 70 | 190 |  | 36,6% | 93 | 192 |  | 48,4 % | 51 | 181 |  | 28,4% | 25 | 207 |  | 12,1% |  |  |
| Bread | 5 | 39 |  | 14,0% | 1 | 24 |  | 2,8 % | 9 | 43 |  | 20,9% | 15 | 38 |  | 39,7% |  |  |
| Cooked vermicelli | 8 | 75 |  | 10,8% | 8 | 89 |  | 8,8 % | 10 | 63 |  | 15,7% | 5 | 68 |  | 6,9% |  |  |
| Corn flour porridge | 15 | 152 |  | 9,8% | 13 | 169 |  | 7,9 % | 18 | 157 |  | 11,2% | 15 | 106 |  | 13,8% |  |  |
| Rice flour porridge | 9 | 151 |  | 5,7% | 5 | 133 |  | 3,7 % | 16 | 183 |  | 9,0% | 4 | 68 |  | 5,2% |  |  |
| Oat porridge (Quacker) | 8 | 148 |  | 5,4% | 3 | 108 |  | 2,8 % | 12 | 146 |  | 8,2% | 17 | 200 |  | 8,6% |  |  |
| Wheat flour donut | 1 | 26 |  | 4,2% | 0,2 | 22 |  | 0,9 % | 1 | 23 |  | 6,0% | 4 | 32 |  | 12,1% |  |  |
| Anagobaka | 9,5 | 258 |  | 3,7% | 6 | 196 |  | 3,3% | 9 | 179 |  | 5,2% | 22 | 1250 |  | 1,7% |  |  |
| Corn dough (To) | 3,1 | 83 |  | 3,7% | 1 | 71 |  | 1,9% | 5 | 94 |  | 5,2% | 5 | 76 |  | 6,9% |  |  |
| Corn flakes | 0,9 | 29 |  | 3,2% | 0,1 | 8 |  | 0,9% | 1 | 17 |  | 6,0% | 4 | 76 |  | 5,2% |  |  |
| Millet flour donut | 0,1 | 13 |  | 0,7% | 0 | 0 |  | 0 | 0 | 0 |  | 0% | 1 | 13 |  | 5,2% |  |  |
| Sorghum porridge | 0,4 | 150 |  | 0,3% | 0 | 0 |  | 0 | 1 | 150 |  | 0,8% | 0 | 0 |  | 0% |  |  |
| Rice porridge | 0,4 | 150 |  | 0,3% | 0 | 0 |  | 0 | 0 | 0 |  | 0% | 3 | 150 |  | 1,7% |  |  |
| Couscous | 0,1 | 60 |  | 0,3% | 0,3 | 60 |  | 0,5% | 0 | 0 |  | 0% | 0 | 0 |  | 0% |  |  |
| **Roots, tubers, starchy foods and derived products** | **43** | **115** | **7,1 %** | **37,6%** | **24** | **105** | **3,6%** | **23,3%** | **47** | **102** | **7,3%** | **45,5%** | **105** | **145** | **18,9%** | **72,4%** | **0.01** | **<0.0001** |
| Cassava semolina (Attieke) | 21 | 101 |  | 20,4% | 7 | 89 |  | 7,9% | 23 | 84 |  | 26,9% | 66 | 128 |  | 51,7% |  |  |
| Mashed potato | 10 | 109 |  | 8,9% | 11 | 110 |  | 9,8% | 7 | 96 |  | 7,5% | 12 | 136 |  | 8,6% |  |  |
| Mashed cassava (placali) | 5 | 113 |  | 4,4% | 1 | 61 |  | 2,3% | 7 | 110 |  | 6,7% | 13 | 187 |  | 6,9% |  |  |
| Mashed sweet potato | 4 | 100 |  | 3,4% | 2 | 119 |  | 1,9% | 5 | 94 |  | 5,2% | 5 | 90 |  | 5,2% |  |  |
| Fried potatoes | 1 | 46 |  | 2,5% | 1 | 100 |  | 0,9% | 2 | 35 |  | 5,2% | 0,1 | 8 |  | 1,7% |  |  |
| Fried plantain | 1 | 40 |  | 1,7% | 0,2 | 50 |  | 0,5% | 1 | 43 |  | 2,2% | 1 | 33 |  | 5,2% |  |  |
| Fried sweet potato | 1 | 32 |  | 1,7% | 0 | 8 |  | 0,5% | 1 | 45 |  | 2,2% | 1 | 27 |  | 5,2% |  |  |
| Mashed yam | 1 | 113 |  | 1,2% | 2 | 115 |  | 1,4% | 1 | 100 |  | 0,8% | 2 | 120 |  | 1,7% |  |  |
| Fried yam | 1 | 152 |  | 0,5% | 0,2 | 48 |  | 0,5 % | 0 | 0 |  | 0% | 4 | 256 |  | 1,7% |  |  |
| **Fish and derived products** | **24** | **48** | **3,9 %** | **49,4%** | **13** | **39** | **2,4%** | **34,9%** | **33** | **53** | **5,0%** | **63,4%** | **39** | **55** | **6,4%** | **70,7%** | **0.01** | **<0.0001** |
| Boiled fatty fish | 14 | 44 |  | 31,7% | 9 | 43 |  | 21,4% | 19 | 45 |  | 43,3% | 18 | 42 |  | 43,1% |  |  |
| Fish | 5 | 37 |  | 13,0% | 2,5 | 27 |  | 9,3% | 8 | 43 |  | 17,9% | 7 | 47 |  | 15,5% |  |  |
| Grilled fatty fish | 5 | 45 |  | 10,6% | 1,5 | 41 |  | 3,7% | 6 | 44 |  | 14,2% | 13 | 48 |  | 27,6% |  |  |
| Boiled lean fish | 0 | 10 |  | 0,3% | 0 | 0 |  | 0% | 0,1 | 10 |  | 0,8% | 0 | 0 |  | 0% |  |  |
| Grilled lean fish | 0 | 20 |  | 0,3% | 0,1 | 20 |  | 0,5% | 0 | 0 |  | 0% | 0 | 0 |  | 0% |  |  |
| **Oils** | **2** | **6** | **2,0 %** | **33,2%** | **1** | **5** | **1,4%** | **28,8%** | **3** | **7** | **2,3%** | **34,3%** | **3** | **7** | **3,2%** | **46,6%** | **0.004** | **0.04** |
| Palmnut Oil | 2 | 6 |  | 33,2% | 1 | 5 |  | 28,8% | 3 | 7 |  | 34,3% | 3 | 7 |  | 46,6% |  |  |
| **Sugar and sugary products** | **4** | **12** | **1,7 %** | **30,0%** | **2** | **11** | **1,0%** | **19,5%** | **5** | **12** | **2,0%** | **40,3%** | **7** | **16** | **3,2%** | **44,8%** | **0.09** | **<0.0001** |
| Sugar | 2 | 8 |  | 23,3% | 1 | 9 |  | 14,9% | 2 | 7,6 |  | 32,1% | 3 | 8 |  | 34,5% |  |  |
| Small biscuit | 1 | 10 |  | 4,9% | 0,4 | 9 |  | 4,2% | 1 | 12 |  | 6,0% | 1 | 9 |  | 5,2% |  |  |
| Medium biscuit | 1 | 27 |  | 2,7% | 0,3 | 28 |  | 0,9% | 1 | 26 |  | 4,5% | 1 | 28 |  | 5,2% |  |  |
| Chocolate spread | 1 | 24 |  | 2,5% | 0,1 | 30 |  | 0,5% | 0,4 | 20 |  | 2,2% | 3 | 25 |  | 10,3% |  |  |
| **Fruits and derived products** | **12** | **79** | **1,4 %** | **15,2%** | **11** | **73** | **1,2%** | **14,4%** | **13** | **97** | **1,5%** | **13,4%** | **15** | **65** | **1,7%** | **22,4%** | **0.20** | **0.25** |
| Banana | 2 | 39 |  | 5,2% | 2 | 32 |  | 4,7% | 1 | 38 |  | 2,2% | 7 | 48 |  | 13,8% |  |  |
| Plantain | 3 | 71 |  | 3,9% | 1 | 67 |  | 1,9% | 5 | 81 |  | 6,0% | 4 | 55 |  | 6,9% |  |  |
| Apple compote | 4 | 95 |  | 3,9% | 5 | 87 |  | 5,6% | 4 | 120 |  | 3,0% | 0 | 0 |  | 0% |  |  |
| Orange | 1 | 65 |  | 2,0% | 2 | 66 |  | 2,3% | 0 | 0 |  | 0% | 3 | 65 |  | 5,2% |  |  |
| Papaya | 1 | 93 |  | 1,0% | 1 | 76 |  | 0,9% | 2 | 110 |  | 1,5% | 0 | 0 |  | 0% |  |  |
| Avocado | 0,4 | 58 |  | 0,7% | 0,4 | 46 |  | 0,9% | 1 | 82 |  | 0,8% | 0 | 0 |  | 0% |  |  |
| Mandarine | 0,1 | 24 |  | 0,5% | 0,2 | 24 |  | 0,9% | 0 | 0 |  | 0% | 0 | 0 |  | 0% |  |  |
| Mango | 1 | 100 |  | 0,5% | 0 | 0 |  | 0% | 2 | 100 |  | 1,5% | 0 | 0 |  | 0% |  |  |
| Apple | 0,2 | 38 |  | 0,5% | 0,1 | 26 |  | 0,5% | 0 | 0 |  | 0% | 0,9 | 51 |  | 1,7% |  |  |
| **Meat, poultry and derived products** | **4** | **38** | **1,2 %** | **10,8%** | **1** | **23** | **0,3%** | **4,7%** | **7** | **47** | **1,9%** | **14,2%** | **10** | **37** | **3,8%** | **25,9%** | **0.19** | **<0.0001** |
| Other Meat | 3 | 34 |  | 7,6% | 1 | 23 |  | 2,8% | 5 | 46 |  | 9,7% | 6 | 27 |  | 20,7% |  |  |
| Chicken | 1 | 37 |  | 3,9% | 0,4 | 21 |  | 1,9% | 2 | 46 |  | 4,5% | 4 | 38 |  | 10,3% |  |  |
| Salami | 0 | 6 |  | 0,5% | 0 | 0 |  | 0% | 0,1 | 6 |  | 1,5% | 0 | 0 |  | 0% |  |  |
| **Vegetables and derived products** | **31** | **77** | **1,2 %** | **40,8%** | **24** | **82** | **0,9%** | **29,8%** | **36** | **73** | **1,2%** | **50,0%** | **46** | **76** | **1,8%** | **60,3%** | **0.69** | **<0.0001** |
| Tomato | 15 | 49 |  | 31,0% | 11 | 51 |  | 21,9% | 24 | 57 |  | 43,1% | 18 | 44 |  | 40,3% |  |  |
| Onion | 5 | 21 |  | 25,3% | 3 | 22 |  | 15,8% | 11 | 25 |  | 43,1% | 6 | 19 |  | 32,8% |  |  |
| Carrot | 7 | 58 |  | 11,6% | 8 | 65 |  | 11,6% | 6 | 51 |  | 12,1% | 6 | 50 |  | 11,2% |  |  |
| Spinach | 2 | 40 |  | 4,9% | 1 | 32 |  | 2,8% | 2 | 47 |  | 5,2% | 4 | 43 |  | 8,2% |  |  |
| Aubergine | 0,5 | 16 |  | 3,4% | 1 | 16 |  | 4,2% | 1 | 18 |  | 3,5% | 0,3 | 12 |  | 2,2% |  |  |
| Onion leaves | 0,1 | 5 |  | 1,2% | 0,1 | 6 |  | 1,9% | 0 | 2 |  | 1,7% | 0 | 0 |  | 0% |  |  |
| Gombo | 0,3 | 29 |  | 1,0% | 0,3 | 54 |  | 0,5% | 0 | 0 |  | 0% | 0,5 | 21 |  | 2,2% |  |  |
| Cassava leaves | 0,2 | 33 |  | 0,7% | 0 | 0 |  | 0% | 1 | 40 |  | 3,5% | 0,1 | 20 |  | 0,8% |  |  |
| Red sorrel (Dah or Bissap) leaves* | 0,6 | 81 |  | 0,7% | 0 | 0 |  | 0% | 0 | 2 |  | 1,7% | 2 | 120 |  | 1,5% |  |  |
| Cucumber | 0,1 | 13 |  | 0,5% | 0 | 0 |  | 0% | 0 | 0 |  | 0% | 0,2 | 13 |  | 1,5% |  |  |
| Squash | 0,0 | 10 |  | 0,5% | 0,1 | 10 |  | 0,9% | 0 | 0 |  | 0% | 0 | 0 |  | 0% |  |  |
| Cabbage | 0,0 | 5 |  | 0,3% | 0 | 0 |  | 0% | 0,1 | 5 |  | 1,7% | 0 | 0 |  | 0% |  |  |
| Courgette | 0,0 | 5 |  | 0,3% | 0 | 5 |  | 0,5% | 0 | 0 |  | 0% | 0 | 0 |  | 0% |  |  |
| Sweet potato leaves | 0,1 | 40 |  | 0,3% | 0 | 0 |  | 0% | 0 | 0 |  | 0% | 0,3 | 40 |  | 0,8% |  |  |
| **Eggs and derived products** | **6** | **41** | **1,0 %** | **15,2%** | **3** | **36** | **0,5%** | **7,9%** | **9** | **41** | **1,3%** | **21,6%** | **12** | **45** | **2,1%** | **27,6%** | **0.34** | **<0.0001** |
| Eggs | 6 | 41 |  | 15,2% | 3 | 36 |  | 7,9% | 9 | 41 |  | 21,6% | 12 | 45 |  | 27,6% |  |  |
| **Pulses and derived products** | **3** | **84** | **0,9 %** | **2,9%** | **2** | **83** | **0,8%** | **2,8%** | **2** | **108** | **1,0%** | **2,2%** | **3** | **62** | **0,7%** | **5,2%** | **0.79** | **0.53** |
| Soy flour | 1 | 114 |  | 1,2% | 2 | 285 |  | 1,4% | 1 | 45 |  | 1,7% | 1 | 80 |  | 1,4% |  |  |
| Mashed beans | 0,2 | 33 |  | 0,7% | 0,1 | 20 |  | 0,5% | 1 | 40 |  | 1,7% | 0,2 | 40 |  | 0,5% |  |  |
| Small beans (lentils) | 0,5 | 100 |  | 0,5% | 0,1 | 20 |  | 0,5% | 2 | 100 |  | 1,7% | 0,5 | 100 |  | 0,5% |  |  |
| Green peas | 0,3 | 70 |  | 0,5% | 2 | 285 |  | 0,5% | 0 | 0 |  | 0% | 1 | 120 |  | 0,5% |  |  |
| **Sauces and condiments** | **12** | **49** | **0,5 %** | **25,6%** | **3** | **33** | **0,1%** | **10,2%** | **19** | **47** | **0,7%** | **40,3%** | **31** | **65** | **1,6%** | **48,3%** | **0.001** | **<0.0001** |
| Aubergine sauce | 5 | 41 |  | 12,5% | 2 | 26 |  | 6,5% | 7 | 40 |  | 18,7% | 13 | 61 |  | 20,7% |  |  |
| Gombo sauce | 3 | 52 |  | 6,4% | 2 | 45 |  | 3,3% | 5 | 54 |  | 9,0% | 7 | 56 |  | 12,1% |  |  |
| Peanut sauce | 1 | 43 |  | 3,0% | 0,1 | 26 |  | 0,5% | 2 | 33 |  | 5,2% | 5 | 65 |  | 6,9% |  |  |
| Palmnut concentrate sauce | 2 | 51 |  | 3,0% | 0 | 0 |  | 0% | 2 | 41 |  | 6,0% | 5 | 72 |  | 6,9% |  |  |
| Djoumgble sauce (dried gombo) | 1 | 47 |  | 2,0% | 0 | 0 |  | 0% | 2 | 46 |  | 4,5% | 2 | 52 |  | 3,5% |  |  |
| Gouagoussou (Aubergine and gombo) sauce | 0,3 | 29 |  | 1,0% | 0,1 | 26 |  | 0,5% | 0,4 | 26 |  | 1,5% | 0,7 | 39 |  | 1,7% |  |  |
| **Beverages** | **9** | **84** | **0,5 %** | **10,3%** | **4** | **67** | **0,2%** | **6,5%** | **6** | **65** | **0,3%** | **9,7%** | **30** | **115** | **1,8%** | **25,9%** | **0.05** | **<0.0001** |
| Orange juice | 6 | 86 |  | 7,4% | 4 | 66 |  | 5,6% | 5 | 61 |  | 7,5% | 21 | 149 |  | 13,8% |  |  |
| Chocolate drink | 2 | 83 |  | 2,7% | 1 | 75 |  | 0,9% | 2 | 113 |  | 1,5% | 9 | 76 |  | 12,1% |  |  |
| Sugar-sweetened beverage | 0 | 15 |  | 0,3% | 0 | 0 |  | 0% | 0,1 | 15 |  | 0,8% | 0 | 0 |  | 0% |  |  |
| **Nuts, seeds and derived products** | **0,4** | **23** | **0,3%** | **2,0%** | **0,3** | **23** | **0,2%** | **1,4%** | **0,3** | **14** | **0,2%** | **2,2%** | **1** | **35** | **0,8%** | **3,4%** | **0.38** | **0.58** |
| Peanut paste | 0,4 | 23 |  | 2,0% | 0,3 | 23 |  | 1,4% | 0,3 | 14 |  | 2,2% | 1 | 35 |  | 3,4% |  |  |

* (*hibiscus sabdaridfa*)
